# *Moringa oleifera* Supplementation as a Natural Galactagogue: A Systematic Review on Its Role in Supporting Milk Volume and Prolactin Levels

**DOI:** 10.3390/foods14142487

**Published:** 2025-07-16

**Authors:** Mohammad Ammar, Giovanni Luca Russo, Almothana Altamimi, Mohammad Altamimi, Mohammed Sabbah, Asmaa Al-Asmar, Rossella Di Monaco

**Affiliations:** 1 Department of Agricultural Sciences, Unit of Food Science and Technology, University of Naples Federico II, 80055 Portici, Italy; mohammedim.ammar@unina.it (M.A.); rossella.dimonaco@unina.it (R.D.M.); 2 Department of Medicine and Surgery, Università di Napoli Federico II, 80133 Naples, Italy; almothana.alt@gmail.com; 3 Department of Nutrition and Food Technology, An-Najah National University, Nablus P4110257, Palestine; m.altamimi@najah.edu (M.A.); m.sabbah@najah.edu (M.S.); 4 Faculty of Science, An-Najah National University, P.O. Box 7, Nablus P4110257, Palestine; a.alasmar@najah.edu; 5 Energy, Water and Food Security Research Center-Agriculture and Food Research Unit, Scientific Centers, An-Najah National University, P.O. Box 7, Nablus P4110257, Palestine

**Keywords:** sustainable diet, prolactin, breastfeeding, postpartum, lactation insufficiency

## Abstract

Breast milk is the optimal nutrition for infants, yet lactation insufficiency remains a common cause of early breastfeeding cessation. *Moringa oleifera* has been traditionally used as a galactagogue due to its rich micronutrient and phytosterol content. This systematic review assessed the effects of *Moringa* leaf supplementation on prolactin levels and breast milk volume in postpartum mothers with lactation insufficiency. A systematic search following PRISMA guidelines, was conducted for randomized controlled trials involving healthy postpartum women supplemented with *Moringa oleifera*. Risk of bias was evaluated using the Cochrane Risk of Bias Tool. Eight studies met the inclusion criteria, with intervention durations ranged from 3 to 10 days. *Moringa* supplementation increased significantly breast milk volume by up to 400 mL/day compared to controls. Serum prolactin levels also rose significantly with a mean increase of 231.72 ng/mL Most studies exhibited low to moderate risk of bias, though one study exhibited high risk due to lack of binding and subjective outcome measurement. *Moringa oleifera* leaf supplementation appears to enhance lactation by increasing milk volume and prolactin levels in postpartum mothers. However, further longer-term studies are needed to establish optimal dosing, sustained effectiveness, and safety.

## 1. Introduction

Breast milk is widely recognized as the ideal source of nutrition for infants, containing a complex composition of macronutrients and bioactive compounds essential for infant growth and immune development [1,2]. Insufficient breast milk production can result from a range of factors including maternal stress, illness, preterm birth, hormonal imbalance, and nutritional deficiencies. Prolactin, a key lactogenic hormone, plays a central role in this process. Secreted by the anterior pituitary in response to nipple stimulation, prolactin initiates and maintains milk secretion by promoting mammary gland development and alveolar function [3]. Most galactagogues (milk booster) exert their effects by enhancing prolactin secretion, which directly correlates with early postpartum breast milk output. However, most available pharmacological galactagogues including domperidone, metoclopramide, and sulpiride can cause adverse effects and may not align with sustainable health goals [3,4]. As such, attention has turned toward non-pharmacological, natural alternatives, including botanical galactagogues like *Moringa oleifera* [5].

*Moringa oleifera* (commonly known as “kelor” or “tree of life”) belonging to the *Moringaceae* family, is considered a nutrient-dense plant due to its rich composition. *Moringa* leaves are rich in proteins, fatty acids (essential and non-essential), amino acids and vitamins (including vitamin A, B1, B2, B3, C, and E) [6], and minerals such as Na, K, Mg, P, Fe, Zn, Cu, Ca, and Mn [7]. Moreover, the plant contains significant amounts of phytochemicals including phenolic compounds, such as flavonoids and tannins, as well as alkaloids and saponins [8]. *Moringa* meets the criteria for sustainable food and diet because it has a positive impact on the environment and contributes to food security and a healthy life. Moreover, its nutritional profile, cultural acceptability, and safety for dietary supplementation have been documented in several populations, including in Zambia, where *Moringa oleifera* leaf powder was well accepted and found safe for short-term use among malnourished girls [9].

*Moringa oleifera* exhibits nutraceutical and pharmacological properties and has been utilized in the prevention and treatment of neuro-dysfunctional diseases, cancers, diabetes, and a range of inflammatory conditions, including rheumatoid arthritis, asthma and inflammatory bowel diseases [10,11]. This beneficial effect is attributed to its nutritional composition, which includes all essential amino acids, key micronutrients, and various bioactive compounds such as polyphenols and phytosterols [12]. These bioactive compounds are associated with increased prolactin levels, thereby enhancing milk production. Phytosterols and steroids function mechanistically by boosting the protoplasmic activity of mammary secretory cells, activating breast secretory neurons directly to enhance milk production, or stimulating the prolactin hormone [13].

*Moringa oleifera* leaves also exhibits antimicrobial, anti-inflammatory, antioxidant, anti-cancer, hepatoprotective, neuroprotective, hypoglycaemic, and blood lipid-lowering activities [14]. Nowadays, it is widely accepted to use natural herbs that help to implement the breast milk production in postpartum lactating mothers [15]. These substances, known as “galactagogues” (pharmaceutical and herbal compounds used to increase lactation), include Shatavari, fenugreek, silymarin, garlic, malunggay, blessed thistle, goat’s rue, anise, black cumin, dill, chasteberry, marshmallow, and fennel [15,16,17]. Safety and efficiency of these products are essential for sustainable milk production, contributing to the long-term health of both mothers and infants [18].

Due to its galactagogues ability, *Moringa oleifera* is known as “Mother’s Best Friend” [6]. Although breast milk is an ideal food for infant growth [19], lactation is a symbiotic process, as lactation defects may originate from the mother, the infant, or both [20,21]. In research on early breastfeeding difficulties, infant’s failure to latch on (40%) was found as the most common reason for early cessation [17]. Moreover, the mother’s perception that the infant is not satisfied with breast milk alone is also reported as the most frequent reason for early breastfeeding cessation [22,23]. In addition, contributing factors included maternal or infant health concerns, such as maternal illness, required medications, or infant health issues, as well as breastfeeding-related challenges like lactation difficulties and problems with milk expression [17,20,24].

Lactation insufficiency after childbirth is a common reason for early breastfeeding termination [25] and can be caused by several conditions. Physiological factors affect breast milk volume, such as abnormal breast anatomy resulting from previous breast surgery, reduction or augmentation of the tissue, and mammary gland hypoplasia (underdevelopment) [16]. Additionally, inadequate stimulation of mammary glands by prolactin and oxytocin hormones can negatively affect the breast milk volume. Under stress conditions, blood flow decreases and in turn, reduces milk secretion due to the lack of supply of oxygen, glucose, fatty acids, and amino acids to the mammary gland [24,26].

Furthermore, certain pathologies can disrupt lactation hormones, directly influencing milk synthesis. These conditions include polycystic ovarian syndrome (PCOS), hyperandrogenism obesity, thyroid disease, and diabetes. In addition, lactose synthesis and insulin signaling play crucial roles in milk production, with gene modulation significantly affecting these physiological processes. Previous research by Lemay et al. (2013) [27] identified significant modulation of key genes involved in these pathways, suggesting a critical interplay between insulin resistance and lactation performance. Among these genes, protein tyrosine phosphatase, receptor type, F (PTPRF) has been proposed as a potential biomarker linking insulin resistance to lactation insufficiency. This finding underscores the importance of insulin signaling in lactogenesis and provides a molecular target for understanding metabolic disorders that affect milk production efficiency [27,28,29,30].

Unlike the earlier narrative review that evaluated the impact of *Moringa oleifera* supplementation during pregnancy, including studies where *Moringa* was administered in various forms, such as leaf powder or as an aqueous extract, in combination with other supplements or as a part of fortified foods [31], those prenatal studies highlighted its effects on maternal hematological profiles, enhanced breast milk production, and improved sociopersonal development in infants. In contrast, this systematic review specifically evaluates postpartum supplementation forms, deliberately excluding food fortification due to the variability of dosage and difficulties in accurately measuring hormonal impacts. Moreover, capsule supplementation ensures accurate dosing, preserves bioactive compounds, and allows for better control over dietary confounders, thereby enabling stronger methodological designs such as placebo use and blinding factors that collectively enhance the validity and comparability of studies and further justify the exclusion of food fortification. The review aims to rigorously assess current evidence supporting *Moringa oleifera* supplementation as a practical, accessible, and sustainable intervention to improve lactation, thereby supporting evidence-based clinical practice and informing public health policies on breastfeeding.

## 2. Materials and Methods

### 2.1. Search Strategy

The Preferred Reporting Items for Systematic Reviews and Meta-Analyses (PRISMA 2020) guidelines were followed throughout this systematic review [32]. We conducted a comprehensive literature search using four databases: PubMed, ScienceDirect, SpringerLink, and Google Scholar. The search was limited to experimental (intervention) studies published from January 2000 to December 2024. Google Scholar was included to identify relevant grey, although duplicate entries and non-peer-reviewed preliminary reports were excluded during the eligibility assessment. The search combined free-text keywords and controlled vocabulary (Medical Subject Headings, MeSH) where applicable. Boolean operators (AND, OR) were used to structure the query logically. The full search strategies for each database, including all search terms and limits applied, are provided in the Supplementary Materials. Two reviewers independently screened titles, abstracts, and full texts against the inclusion criteria. Discrepancies were resolved through discussion or by involving a third reviewer. The primary outcome assessed was breast milk volume, and secondary outcome was prolactin hormone level. Breast milk production was measured using standardized pumping methods and recorded in millimetres per day (mL/day), while prolactin was measured from blood serum using special kit/microplate reader (ng/mL).

### 2.2. Study Selection and Patient Population

**Figure 1:**
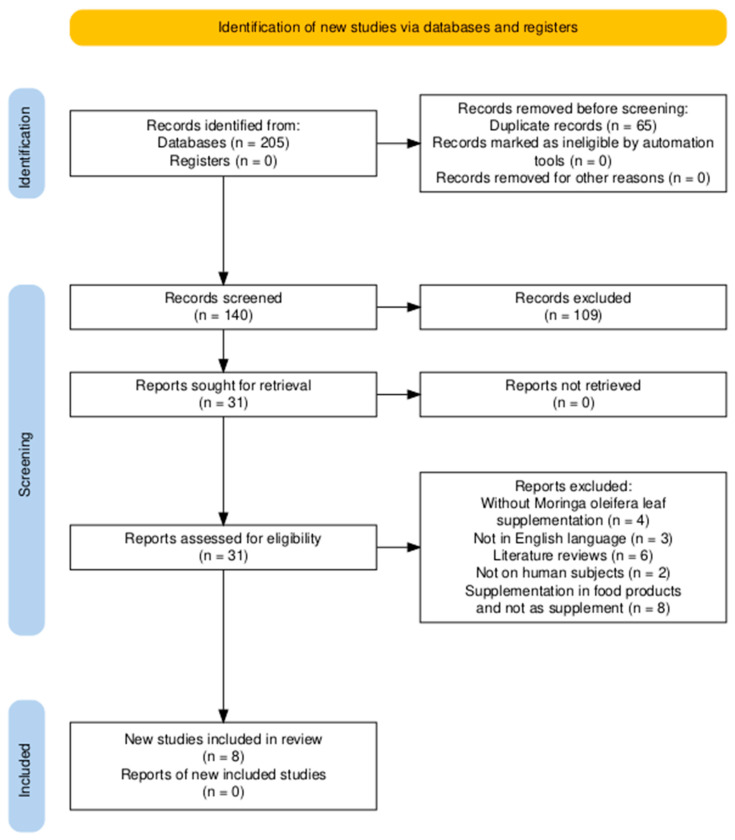
PRISMA (Preferred Reporting Items for Systematic Reviews and Meta-Analyses) flow diagram for studies retrieved through the searching and selection process.

The screening of abstracts and titles was the preliminary stage of the research, followed by checking full-text studies that met the following selection criteria: (1) The research was conducted on humans; (2) The research involved a postpartum lactating mother with normal delivery within six months; (3) The research involved a control group; and (4) There was a treatment or intervention group that focused on *Moringa* leaves extract capsule/supplementation. The exclusion criteria used in this research included articles without original research or a protocol, breastfeeding mothers, available full text, and a comparison group as well as those written in another language than English. The flow chart representing screening process is shown in Figure 1.

Participants, intervention, comparators, outcomes, and study design (PICOS) standards are shown in Table 1.

### 2.3. Keywords Co-Occurrence Analysis

Measurements of the quality and quantity of the scientific production were accomplished using VOS Viewer science mapping software tool (version 1.6.20). Previously, selected data for VOS Viewer supported file types (Web of Science and Elsevier Scopus) were exported to Microsoft 365 Excel and then elaborated. From the database, 804 keywords were extrapolated, but for the analysis, the occurrence threshold was set at a minimum of 5 between all of the documents, obtaining 42 selected keywords. This assisted in contextualizing the current systematic review within broader scientific discourse.

### 2.4. Eligibility Assessment and Data Extraction

Two of the team members separately assessed the eligibility of article to be included in the final data extraction. A third member of the team was involved when discrepancies arose. From each article, we gathered the following information: author (s) and publication year, sample size and characteristics of the participants, research methodology, duration of intervention of taking *Moringa* leaves extract/capsule supplementation, the design of the study, and the main results. Before being reviewed, papers were screened in a three phase-selection process. Studies that did not meet the inclusion criteria solely based on their title were excluded in the first phase. A second phase involved screening the abstracts and excluding studies that didn’t meet the inclusion criteria. To standardize data collection, all data extraction was conducted independently using a data extraction form.

### 2.5. Risk of Bias

Studies were tested for risk of bias using RoB 2: a revised tool for assessing risk of bias in randomised trials [33] and the ROBINS-I tool for non-randomized studies. The studies were assessed for the following sources of bias: bias arising from the randomisation process, bias due to deviations from intended interventions, bias due to missing outcome data, bias in measurement of the outcome, and bias in selection of the reported result. Discrepancies were resolved by consensus, or by consulting a third reviewer when necessary. Overall risk was classified as ‘low,’ ‘some concerns,’ or ‘high’ based on consensus among the reviewers.

## 3. Results

### 3.1. Description of Selected Trials

In the initial database search, a total of 185 papers were identified, based on the predefined inclusion and exclusion criteria. After the removal of duplicate publications and exclusion of irrelevant articles, eight articles [7,24,34,35,36,37,38,39] were selected and included in the final analysis as a consequence of this methodology.

The characteristics of the included studies are summarised in Table 2. Participants were healthy postpartum mothers, explicitly free of chronic metabolic or endocrine diseases, and had uncomplicated postpartum recoveries, clinically defined by the absence of postpartum hemorrhage, infection, or breastfeeding-related complications. Additionally, they were willing to breastfeed exclusively and were not taking herbs, breastfeeding supplements, or any type of medication. The mothers’ ages in these studies ranged from 18 to 45 years. Furthermore, all postpartum mothers were randomly assigned to receive either a daily *Moringa* leaf supplement or a placebo. Among the eight articles reviewed, the duration of the intervention varied from 3 to 10 days, except for one study that lasted for a month. Reported outcomes included breast milk volume, prolactin hormone levels, and milk fat percentage.

**Table 2:** General characteristics and risk of bias assessment of included studies.

Type of Study	Population	Intervention and Supplements	Procedure	Outcomes	Overall RoB	Reference
**Pretest and post-test group design**	20 postpartum mothers (age not reported).	800 mg capsule, twice a day, for 1 month (ethanolic extract + *Moringa* powder, 1:4 ratio).	Taking the breast milk volume and after two treatments of acupressure and two capsules per day for 1 month visit in observation of breast milk volume.	Increase in breast milk volume (>400 mL/day).	Serious	[7]
**Double-blind, randomized controlled trial.**	68 postpartum mothers (aged 20–45 years).	Capsule of 250 mg every 12 h from day 3 postpartum (commercial formulation).	Instructing mothers to use a standardized breast pump to pump their breasts every 4 h. The volume was measured with standardized containers and recorded in study logbook provided by the research team.	Increase in breast milk volume (152–176 mL/day).	Low	[24]
**Single-blind Randomized Controlled Trial.**	82 postpartum mothers (aged 18–38 years).	2 capsules of 350 mg/day from day 8 postpartum.	Milk collected using a breast pump. The expressed milk was immediately transferred to a standard container, and the amount of milk was recorded.	Augmentation of breast milk volume (up to 245 mL/day).	low	[34]
**Randomized, double-blind, placebo-controlled trial.**	(88 postpartum mothers (>18 years old).	1 capsule (450 mg), twice a day for 3 days (commercial leaf powder).	Taking the infant’s weight on the third postpartum day (48–72 h). The difference in weight in grams was then converted to the volume of breast milk in milliliters (1 g = 1 mL approximation).	30% increase in breast milk volume (123.8 ± 84.9 mL/day).	Low	[35]
**Quasi-Experimental study with Non Equivalent control group design**	Breastfeeding postpartum mothers (30 postpartum mothers, aged 20–35 years).	250 mg capsule, once daily 30 min before breastfeeding for 14 days.	Taking a blood sample for both groups, and measuring prolactin hormone levels by a Microplate Reader.	Increased level of prolactin hormones (231.72 ng/mL).	Some concern	[36]
**Quasi-Experimental study with Non Equivalent control group design**	36 postpartum mothers (aged 27–30 years).	250 mg capsule twice a day for 2 weeks (leaf powder capsules).	Measuring breast milk fat was done through laboratory measurments with the Soxhlet method, which is considered an indicator for increasing breast milk production.	Increased breast fat milk (from 4% to 4.5%)	Some concern	[37]
**Quasi-experimental with pre/post-test control group design**	Postpartum mothers, 7–10 days postpartum, exclusively breastfeeding.	Standardized *Moringa* oleifera extract tablets, 2 × 2 capsules/day for 30 days.	Infant weight was measured at baseline and at 30 days postpartum to assess breast milk adequacy indirectly.	Increased infant weight as a proxy for milk production.	Moderate	[38]
**Quasi-experimental with combined intervention**	40 postpartum mothers, age not reported.	*Moringa* capsule 650 mg daily + acupressure for 10 days.	Prolactin measured via ELISA; infant weight monitored over 10 days.	Increased prolactin levels and infant weight.	Serious	[39]

### 3.2. Database Analysis Results

**Figure 2:**
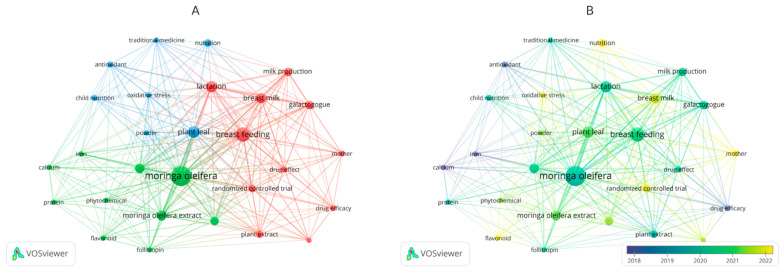
Keyword co-occurrence networks of *Moringa oleifera* in the context of lactation and maternal health. (**A**) Nodes are color-coded by thematic clusters. (**B**) The network is overlaid with a temporal gradient (2018–2023) to illustrate the evolving focus on *Moringa oleifera*.

A word cloud related to searched keywords is shown in Figure 2. The font size of the given keywords indicates the number of times they appear in the literature records.

Figure 2A,B illustrates the keyword co-occurrence networks for *Moringa oleifera*–related research. In both subfigures, *Moringa oleifera* is a central node, underscoring its pivotal role in recent scientific discourse. High-connectivity terms such as “breastfeeding,” “breast milk,” and “lactation” highlight a growing body of work linking *Moringa oleifera* to improve milk production and maternal well-being. Notably, terms like “child nutrition,” “antioxidant,” and “oxidative stress” suggest that interest extends beyond lactation outcomes, exploring broader nutritional benefits that may support infant development. Keywords such as “randomized controlled trial” and “drug effect” underscore an emerging methodological rigor aimed at elucidating *Moringa oleifera*’s therapeutic efficacy. The color gradient in panel B reveals an intensified focus on the galactagogue effects of *Moringa oleifera* over the past few years, mirroring the rise in clinical and scientific interest. Crucially, these networks highlight the novelty of *Moringa oleifera* in addressing breastfeeding challenges. While various herbal galactagogues have long been studied, *Moringa oleifera* stands out for its broad nutrient spectrum and apparent safety profile, offering a promising intervention for postpartum women who struggle with lactation insufficiency. This heightened interconnectedness of terms suggests rapidly expanding evidence base and underscores the need for further high-quality, large-scale trials. Ultimately, the keyword co-occurrence patterns strengthen the case for *Moringa oleifera* as a novel, multifaceted approach to improving lactational outcomes and advancing maternal–infant nutrition.

### 3.3. Outcomes on the Milk Volume and Prolactin

All studies consistently reported beneficial effects of *Moringa* leaf supplementation on breast milk production, with increased volumes ranging from 135 mL/day to 400 mL/day compared to placebo groups [7,24,34,35]. Moreover, prolactin levels showed a significant increase following supplementation, with one study reporting a rise from a baseline mean of 152.75 ± 66.99 ng/mL in the control group to 231.72 ± 60.45 ng/mL post-intervention, a statistically significant difference (*p* = 0.002) [36]. In addition, a significant improvement in milk fat content (range: 4.0–4.5%, *p* = 0.002) was reported by one randomized controlled trial [37], clearly highlighting the potential nutritional enhancement associated with *Moringa* supplementation. The statistical methods employed for analyzing outcomes varied among studies, detailed methodological descriptions are systematically provided in Table 2.

### 3.4. Risk of Bias Results

The Risk of Bias assessment was systematically conducted for each included study according to RoB2 criteria, evaluating the following domains: randomization process, deviations from intended interventions, missing outcome data, outcome measurement accuracy, and selective reporting. Three studies [24,34,35] demonstrated a low risk of bias, indicating no significant methodological concerns across all assessed domains; therefore, their findings are considered reliable. Two studies [36,37] raised “some concerns”, due to minor methodological uncertainties, as at least one domain indicated potential bias, reducing confidence slightly in its conclusions. Conversely, two studies [7,39] were categorized as “serious” in terms of RoB, reflecting substantial methodological concerns in multiple domains or at least one critical domain, significantly lowering confidence in the reliability of its findings. The results of selected studies were summarized comprehensively in Table 2.

Across all studies, the most consistent outcome was an increase in breast milk volume, with some studies also reporting elevated serum prolactin levels or increased infant weight gain. However, none of the studies provided detailed phytochemical characterizations of the *Moringa* preparations. Most studies utilized commercially available leaf powder capsules, and no specific data were given regarding active compound concentrations or extraction profiles. Additionally, intervention dosages and durations varied widely across studies, ranging from 250 mg to 800 mg daily and 3 days to 1 month, complicating direct comparisons. Despite these variations, the overall trend suggests that *Moringa oleifera*, when administered in controlled settings, may offer a beneficial, natural approach to supporting lactation, though further standardized, high-quality trials are warranted to confirm optimal dosing strategies and long-term efficacy.

## 4. Discussion

The findings of this systematic review highlight the potential role of *Moringa oleifera* supplementation in enhancing breast milk volume, prolactin levels, and overall maternal health during the postpartum period. Despite the limited number of eligible studies (*n* = 6) all reported positive effects on breast milk volume, prolactin levels or milk composition, indicating potential clinical benefits of *Moringa* supplementation. Overall, we can presume that encapsulated *Moringa* supplements provide a convenient and effective means for postpartum mothers to access these benefits. However, while *Moringa oleifera* leaves were consistently administered in encapsulated form across all included studies, there was considerable heterogeneity, and in some cases lack of reporting, regarding the specific type of extract and encapsulation process. Most studies employed powdered *Moringa* leaf directly, while only one [7] used a hydroalcoholic extract subjected to maceration, rotary evaporation, and freeze-drying. Furthermore, the capsule shell material and encapsulation methods were not disclosed, representing a limitation in reproducibility and formulation consistency. Moreover, the included studies employed diverse dosages (250–800 mg/day) and intervention durations (3 days to 1 month), reflecting the lack of consensus on optimal use [34].

The galactagogue effect of *Moringa oleifera* is likely mediated through its phytosterols and polyphenols, which may stimulate prolactin production or directly activate mammary secretory pathways. Sulistiawati’s findings [36] provide a mechanistic anchor, while the increased milk fat content observed by Yuliastuti et al. [37] expands the therapeutic scope to qualitative improvements in milk. Additionally, the rich nutritional profile of *Moringa oleifera*, including essential amino acids, vitamins, minerals, and phytosterols, likely supports maternal metabolism and milk synthesis during high demand lactation period [15,40]. Wahidah et al. [39] reported significantly increased prolactin levels and infant weight gain following a combined intervention involving *Moringa* supplementation and acupressure. Although the presence of a secondary intervention limits attribution solely to *Moringa*, the significant hormonal and clinical improvements remain noteworthy. Conversely, Septadina and Murti [41] demonstrated that supplementation with *Moringa* extract alone, resulted in increased infant weight over 30 days, serving as an indirect but clinically meaningful proxy for improved milk supply.

Although the total number of articles included in this review was limited, the findings contribute meaningfully to the existing body of knowledge about the potential of *Moringa* leaves as a plant-based supplement for lactation. Additionally, *Moringa* offers an eco-friendly, cost-effective solution aligned with the principles of a sustainable diet, making it especially beneficial in resource-limited settings [15]. Nevertheless, all studies included in this review consistently reported positive effects of *Moringa oleifera* supplementation on lactation outcomes. This adds valuable insight into the natural management of lactation insufficiency a primary cause of breastfeeding cessation, which affects up to 60% of mothers in the early postpartum period.

In fact, inadequate milk production is regularly noted and physicians typically treat this common clinical problem by prescription medications and other medicines to boost milk production, such as galactagogues [16]. 

**Figure 3:**
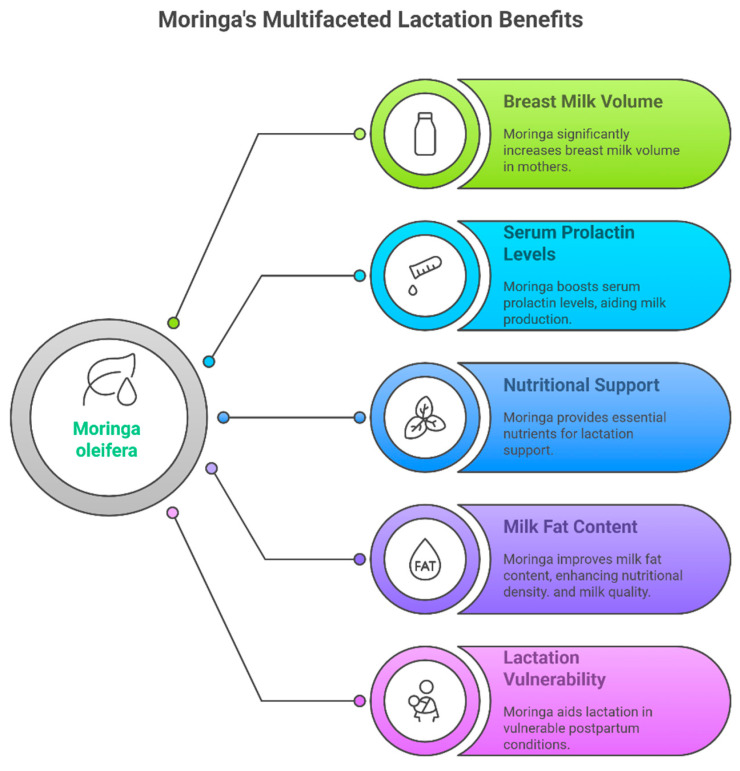
A conceptual overview illustrating lactation benefits of *Moringa oleifera* leaf supplementation.

A summary of the findings on the effects of *Moringa*’s benefits in lactation period has been shown in Figure 3.

A key strength of the reviewed literature is the absence of reported adverse effects in both mothers and infants across all trials. No gastrointestinal disturbances, allergic reactions, or neonatal complications were noted. Without side effects or health complications, *Moringa oleifera* leaf extract plays a positive role in enhancing the lactagogum effect, which stimulates the hormones prolactin. The lactogenic benefits are attributed to its nutritional composition, which include proteins (containing all essential amino acids), vitamins, minerals, and bioactive compounds like phytosterols. These compounds promote mammary gland secretion, either by acting on nerve endings in the mammary glands, thus increasing milk production, or by directly stimulating prolactin, which affects alveolar epithelial cells [41,42,43]. As illustrated in Figure 3, *Moringa*’s benefits extend beyond milk volume to include improvements in prolactin levels, milk composition (fat content), nutritional support, and effectiveness in vulnerable lactation contexts. In addition, our review findings are consistent with prior research that reported similar positive effects of *Moringa oleifera* leaves as a natural supplement on prolactin hormone levels and breast milk volume [38,42,44]. This alignment with existing literature reinforces the credibility of our conclusions and supports the growing consensus around *Moringa oleifera*’s efficacy as a galactagogue.

All of these factors contribute to an increase in breast milk volume and lactation in postpartum lactating mothers, resulting in sufficient milk supply to support infant nutrition, ensuring adequate milk supply for infants and promoting optimal infant growth and weight [7,24].

## 5. Conclusions

Overall, the evidence from the included studies indicates that the supplementation of *Moringa oleifera* leaf during the postpartum period helps increase breast milk supply both quantitatively and qualitatively. Despite the limited number of included studies, the alignment of findings across all eight studies, including those with varying methodologies and populations, reinforces the credibility of our conclusions and supports a growing scientific consensus around the efficacy of *Moringa oleifera* as a natural galactagogue. The consistency in findings strengthens the evidence that *Moringa oleifera* serves as an effective, accessible, and safe galactagogue. Its use may support natural breastfeeding practices, improve maternal and infant health outcomes, and reduce reliance on infant formula. Consequently, this may reduce the likelihood of early breastfeeding cessation, and encourage the use of plant-based interventions aligned with sustainable health and nutrition strategies.

## 6. Future Perspectives

Among the eight studies included in this review, only one trial measured hormonal biomarkers, specifically serum prolactin. No studies evaluated other key hormones involved in lactation physiology such as oxytocin, estrogens, or thyroid hormones. This limits the mechanistic understanding of *Moringa*’s effects on the broader endocrine regulation of lactation. Future clinical trials should incorporate expanded hormonal panels, including oxytocin and estrogen, to better elucidate the neuroendocrine pathways influenced by *Moringa* supplementation.

Other future research should aim to validate the efficacy of *Moringa oleifera* supplementation in promoting lactation through robust, well-designed human studies across diverse populations. Extended follow-up investigations are warranted to assess long-term maternal and infant health outcomes. Further exploration of the physiological pathways and active constituents involved in lactogenic activity could provide insight into underlying biological mechanisms. Comparative evaluations with established galactagogues may elucidate relative benefits and risks. Moreover, refining parameters such as preparation method, effective concentration, and supplementation period would help guide consistent and evidence-based application.

## 7. Limitations

This systematic review is limited primarily by the small number of eligible clinical trials and the methodological variability across studies. Although all included studies reported positive effects of *Moringa oleifera* supplementation on lactation outcomes, only two were randomized controlled trials with double-blind designs, while the remaining employed quasi-experimental or pretest–posttest approaches, limiting the overall strength of evidence.

A specific limitation concerns the hormonal mechanism underpinning *Moringa*’s effects. Only one study [36] assessed serum prolactin levels, and its findings, while statistically significant, must be interpreted cautiously.

Furthermore, across the selected studies there was heterogeneity in the form and dose of *Moringa* supplementation, ranging from 250 mg to 1600 mg/day, with intervention durations from 3 days to 1 month. None of the studies evaluated oxytocin or other relevant hormones beyond prolactin, which limits mechanistic understanding. Additionally, some of the included studies were published in national journals not indexed in major international databases, while in other studies, the full text was available only in non-English languages, potentially restricting broader accessibility and independent replication.

## Figures and Tables

**Table 1:** Participants, intervention, comparators, outcomes (PICOs), and study design standards for exclusion and inclusion of articles.

	Inclusion	Exclusion
**Population**	Breastfeeding postpartum mothers, healthy mothers, and healthy infants, do not take medication, on humans.	Non-breastfeeding mothers, pregnancy, mothers with chronic diseases, chorioamnionitis, taking any medication, mothers with breast anomalies, mothers of infants with neonatal illness and congenital anomalies and postpartum mothers with complications (bleeding infection), or experiments conducted on animals. Not exclusively breastfeeding.
**Intervention**	Capsules or tablets supplementation with *Moringa* powdered leaf extract.	No supplementation with *Moringa* leaf extract powdered capsules.
**Control**	Treatment (*Moringa* leaf extract), control (Placebo).	No comparison.
**Outcome**	Increases the volume of milk and prolactin.	Poor procedure or no clear findings.
**Study design**	Interventional trial (controlled randomized and uncontrolled (pre-test post-test).	Not related, no-clear finding.

## Data Availability

No new data were created or analyzed in this study. Data sharing is not applicable to this article.

## Supplementary Materials

The following supporting information can be downloaded at: https://www.mdpi.com/article/10.3390/foods14142487/s1.

## Abbreviations

The following abbreviations are used in this manuscript:

PCOS	Polycystic Ovarian Syndrome
PTPRF	Protein Tyrosine Phosphatase, Receptor type, F
PRL	Peptide hormone Prolactin
LTH	Luteotropin
PRISMA	Preferred Reporting Items for Systematic Reviews and Meta-Analyses
PICOs	Participants, Intervention, Comparators, Outcomes
RoB	Risk of Bias

## References

[1] Goksugur S.B., Karatas Z. (2014). Breastfeeding and Galaktogogoneus Agents. Acta Med. Anatolia.

[2] Hill P.D., Aldag J.C., Demirtas H., Naeem V., Parker N.P., Zinaman M.J., Chatterton R.T. (2009). Association of Serum Prolactin and Oxytocin with Milk Production in Mothers of Preterm and Term Infants. Biol. Res. Nurs..

[3] Zuppa A.A., Sindico P., Orchi C., Carducci C., Cardiello V., Catenazzi P., Romagnoli C. (2010). Safety and efficacy of galacto-gogues: Substances that induce, maintain, and increase breast milk production. J. Pharm. Pharm. Sci..

[4] McBride G.M., Stevenson R., Zizzo G., Rumbold A.R., Amir L.H., Keir A.K., Grzeskowiak L.E. (2021). Use and Experiences of Galactagogues While Breastfeeding Among Australian Women. PLoS ONE.

[5] King J., Raguindin P.F., Dans L.F. (2013). *Moringa oleifera* (Malunggay) as a Galactagogue for Breastfeeding Mothers: A Systematic Review and Meta-Analysis of Randomized Controlled Trials. Philipp. J. Pediatr..

[6] Egbuna C. (2015). *Moringa oleifera* “The Mother’s Best Friend”. Int. J. Nutr. Food Sci..

[7] Renityas N.N. (2018). The Effectiveness of Moringa Leaves Extract and Cancunpoint Massage towards Breast Milk Volume on Breastfeeding Mothers. J. Ners Kebidanan.

[8] Abalaka M.E., Daniyan S.Y., Oyeleke S.B., Adeyemo S.O. (2012). The antibacterial evaluation of *Moringa oleifera* leaf extracts on selected bacterial pathogens. J. Microbiol. Res..

[9] Barichella M., Pezzoli G., Faierman S.A., Raspini B., Rimoldi M., Cassani E., Cereda E. (2019). Nutritional Characterisation of Zambian *Moringa oleifera*: Acceptability and Safety of Short-Term Daily Supplementation in a Group of Malnourished Girls. Int. J. Food Sci. Nutr..

[10] Oguntibeju O.O., Aboua G.Y., Omodanisi E.I. (2020). Effects of *Moringa oleifera* on Oxidative Stress, Apoptotic, and Inflammatory Biomarkers in Streptozotocin-Induced Diabetic Animal Model. S. Afr. J. Bot..

[11] Klimek-Szczykutowicz M., Gaweł-Bęben K., Rutka A., Blicharska E., Tatarczak-Michalewska M., Kulik-Siarek K., Szopa A. (2024). *Moringa oleifera* (Drumstick Tree)—Nutraceutical, Cosmetological and Medicinal Importance: A Review. Front. Pharmacol..

[12] Divya S., Pandey V.K., Dixit R., Rustagi S., Suthar T., Atuahene D., Shaikh A.M. (2024). Exploring the Phytochemical, Pharmacological and Nutritional Properties of *Moringa oleifera*: A Comprehensive Review. Nutrients.

[13] Raguindin P.F.N., Dans L.F., King J.F. (2014). *Moringa oleifera* as a galactagogue. Breastfeed. Med..

[14] Kasiri K., Heidari-Soureshjani S., Pocock L. (2017). Medicinal plants’ effect on prolactin: A systematic review. Middle East J. Fam. Med..

[15] Bazzano A.N., Hofer R., Thibeau S., Gillispie V., Jacobs M., Theall K.P. (2016). A review of herbal and pharmaceutical galactagogues for breastfeeding. Ochsner J..

[16] Foong S.C., Tan M.L., Foong W.C., Marasco L.A., Ho J.J., Ong J.H. (2020). Oral galactagogues (natural therapies or drugs) for increasing breast milk production in mothers of non-hospitalised term infants. Cochrane Database Syst. Rev..

[17] Odom E.C., Li R., Scanlon K.S., Perrine C.G., Grummer-Strawn L. (2013). Reasons for earlier than desired cessation of breastfeeding. Pediatrics.

[18] Pareek A., Pant M., Gupta M.M., Kashania P., Ratan Y., Jain V., Chuturgoon A.A. (2023). *Moringa oleifera*: An Updated Comprehensive Review of Its Pharmacological Activities, Ethnomedicinal, Phytopharmaceutical Formulation, Clinical, Phytochemical, and Toxicological Aspects. Int. J. Mol. Sci..

[19] Gatti L. (2008). Maternal Perceptions of Insufficient Milk Supply in Breastfeeding. J. Nurs. Scholarsh..

[20] Amir L.H. (2006). Breastfeeding: Managing ‘Supply’ Difficulties. Aust. Fam. Physician.

[21] Neifert M., Bunik M. (2013). Overcoming Clinical Barriers to Exclusive Breastfeeding. Pediatr. Clin. N. Am..

[22] Gianni M.L., Bettinelli M.E., Manfra P., Sorrentino G., Bezze E., Plevani L., Mosca F. (2019). Breastfeeding Difficulties and Risk for Early Breastfeeding Cessation. Nutrients.

[23] Li R., Fein S.B., Chen J., Grummer-Strawn L.M. (2008). Why Mothers Stop Breastfeeding: Mothers’ Self-Reported Reasons for Stopping During the First Year. Pediatrics.

[24] Estrella M.C.P., Mantaring J.B.V., David G.A. (2000). A Double-Blind, Randomized Controlled Trial on the Use of Malunggay (*Moringa oleifera*) for Augmentation of the Volume of Breastmilk Among Non-Nursing Mothers of Preterm Infants. Philipp. J. Pediatr..

[25] El Sohaimy S.A., Hamad G.M., Mohamed S.E., Amar M.H., Al-Hindi R.R. (2015). Biochemical and Functional Properties of *Moringa oleifera* Leaves and Their Potential as a Functional Food. Glob. Adv. Res. J. Agric. Sci..

[26] Ziomkiewicz A., Babiszewska M., Apanasewicz A., Piosek M., Wychowaniec P., Cierniak A., Wichary S. (2021). Psychosocial Stress and Cortisol Stress Reactivity Predict Breast Milk Composition. Sci. Rep..

[27] Lemay D.G., Ballard O.A., Hughes M.A., Morrow A.L., Horseman N.D., Nommsen-Rivers L.A. (2013). RNA Sequencing of the Human Milk Fat Layer Transcriptome Reveals Distinct Gene Expression Profiles at Three Stages of Lactation. PLoS ONE.

[28] Berens P., Labbok M., Academy of Breastfeeding Medicine (2015). ABM Clinical Protocol #13: Contraception During Breastfeeding, Revised 2015. Breastfeed. Med..

[29] Brownell E.A., Fernandez I.D., Howard C.R., Fisher S.G., Ternullo S.R., Buckley R.J., Dozier A.M. (2012). A Systematic Review of Early Postpartum Medroxyprogesterone Receipt and Early Breastfeeding Cessation: Evaluating the Methodological Rigor of the Evidence. Breastfeed. Med..

[30] Hoover K.L., Barbalinardo L.H., Platia M.P. (2002). Delayed Lactogenesis II Secondary to Gestational Ovarian Theca Lutein Cysts in Two Normal Singleton Pregnancies. J. Hum. Lact..

[31] Rotella R., Soriano J.M., Llopis-González A., Morales-Suarez-Varela M. (2023). The Impact of *Moringa oleifera* Supplementation on Anemia and Other Variables During Pregnancy and Breastfeeding: A Narrative Review. Nutrients.

[32] Moher D., Liberati A., Tetzlaff J., Altman D.G., The PRISMA Group (2009). Preferred Reporting Items for Systematic Reviews and Meta-Analyses: The PRISMA Statement. Ann. Intern. Med..

[33] Sterne J.A.C., Savović J., Page M.J., Elbers R.G., Blencowe N.S., Boutron I., Cates C.J., Cheng H.Y., Corbett M.S., Eldridge S.M. (2019). RoB 2: A Revised Tool for Assessing Risk of Bias in Randomised Trials. BMJ.

[34] Espinosa-Kuo C.L. (2005). A Randomized Controlled Trial on the Use of Malunggay (*Moringa oleifera*) for Augmentation of the Volume of Breastmilk Among Mothers of Term Infants. Filip. Fam Physician.

[35] Fungtammasan S., Phupong V. (2022). The Effect of *Moringa oleifera* Capsule in Increasing Breast Milk Volume in Early Postpartum Patients: A Double-Blind, Randomized Controlled Trial. Eur. J. Obstet. Gynecol. Reprod. Biol. X.

[36] Sulistiawati Y., Suwondo A., Hardjanti T.S., Soejoenoes A., Anwar M.C., Susiloretni K.A. (2017). Effect of *Moringa oleifera* on level of prolactin and breast milk production in postpartum mothers. Belitung Nurs. J..

[37] Yuliastuti S., Kuntjoro T., Sumarni S., Supriyana W.M. (2018). KELOR (*Moringa oleifera*) as an alternative in increasing breast milk production. J. Med. Sci. Clin. Res..

[38] Kristiyanti R., Khuzaiyah S., Chabibah N., Khanifah M. (2020). Effectiveness of Moringa oleifera Extract to Increase Breastmilk Production in Postpartum Mothers with Food Restriction. Proceedings of the 1st Borobudur International Symposium on Humanities, Economics and Social Sciences (BIS-HESS 2019).

[39] Wahidah N., Eko Ningtyas E.A., Latifah L. (2023). Effect of the Combination of Acupressure and Moringa oleifera Extract Consumption on Elevating Breast Milk Production and Adequacy in Lactating Mothers. J. Matern. Child Health.

[40] Mahaveerchand H., Abdul Salam A.A. (2024). Environmental, Industrial, and Health Benefits of *Moringa oleifera*. Phytochem. Rev..

[41] Septadina I.S., Murti K. (2018). Effects of Moringa Leaf Extract (*Moringa oleifera*) in Breastfeeding. Sriwijaya J. Med..

[42] Marsiami A.S., Puspariny C. (2024). The Effectiveness of Moringa Leaf Jelly on Mother’s Prolactin Level and Baby’s Outcome. Int. J. Public Health Sci..

[43] Nurachma E., Lushinta L., Puspitaningsih R., Sholikah I. (2024). The Effect of Moringa Pudding on Increasing Breast Milk for Postpartum Mothers. Asian J. Eng. Soc. Health.

[44] Yasin Z., Nawawi A., Wibowo A., Nadhiroh S.R., Devy S.R. (2024). Effects of Moringa oleifera on increasing breast milk in breastfeeding mothers with stunting toddlers in rural Batang-Batang District, Indonesia. Afr. J. Reprod. Health.

