# Supplementary material for: Moringa oleifera Supplementation as a Natural Galactagogue: A Systematic Review on Its Role in Supporting Milk Volume and Prolactin Levels
Source: Foods. 2025 Jul 16;14(14):2487. doi: 10.3390/foods14142487 (PMC12294722; doi:10.3390/foods14142487)
Supplement: Supplementary file 1 [file foods-14-02487-s001.zip › foods-3682201-supplementary.pdf]

## Supplementary Material

### Full Search Strategy

Search Period: January 2000 – December 2024

Databases Searched: PubMed, ScienceDirect, SpringerLink, Google Scholar

Search Date Range: Final search conducted on 05/07/2025

### 1. PubMed Search Strategy (including MeSH terms)

Search Query Used:

("Moringa oleifera"[Mesh] OR "Moringa extract"[All Fields] OR "Kelor leaf extract"[All Fields])
AND
("Lactation"[Mesh] OR "Breast Feeding"[Mesh] OR "Lactating mothers"[All Fields] OR "Postpartum mothers"[All Fields])
AND
("Prolactin"[Mesh] OR "Prolactin level"[All Fields])
AND
("Dietary Supplements"[Mesh] OR “Tablets” OR “Capsule” OR "Supplementation"[All Fields])

- Filters Applied: Humans, Clinical Trials, English Language

- Fields Searched: Title/Abstract, MeSH terms

- Last search conducted: 05/07/2025

- Notes: No date or age restrictions applied.

### 2. ScienceDirect Search Strategy

Search Query Used:

("Moringa oleifera" OR "Moringa extract" OR "Kelor leaf") AND
("Lactation" OR "Breastfeeding" OR "Prolactin") AND
("Supplementation" OR "Capsules" OR “Tablets) AND
("Postpartum" OR "Lactating mother")

- Search limited to Research Articles and Clinical Trials

- Filters: 2000–2024; English language

### 3. SpringerLink Search Strategy

Search Query Used:

("Moringa oleifera" AND "lactation" AND "prolactin") OR
("Moringa supplementation" AND "breastfeeding") OR
("Moringa capsules" AND "postpartum women")

- Applied filters: Subject = Life Sciences, Medicine & Public Health; Articles only; Published between 2000–2024

## 4. Google Scholar Search Strategy

Search Query Used:

"moringa supplementation" AND ("prolactin" OR "breast milk") AND ("postpartum mothers" OR "lactating women")

- Manual screening applied for grey literature

- Inclusion: Thesis documents, government reports, conference proceedings if relevant

- Exclusion: Preprints, non-peer-reviewed reports, duplicates

Search Outcome Summary:

- Total results retrieved: 205

- Duplicates removed: 65

- Titles and abstracts screened: 140

- Full-text assessed for eligibility: 31

- Final studies included: 8
